# XENON in medical area: emphasis on neuroprotection in hypoxia and anesthesia

**DOI:** 10.1186/2045-9912-3-4

**Published:** 2013-02-01

**Authors:** Ecem Esencan, Simge Yuksel, Yusuf Berk Tosun, Alexander Robinot, Ihsan Solaroglu, John H Zhang

**Affiliations:** 1 Koc University School of Medicine, Rumelifeneri Yolu, Sariyer-Istanbul, 34450, Turkey; 2 Université Lorraine Faculté de Médecine, 9 Avenue de la Forét de Haye, Vandoeuvre-lés-Nancy, 54505, France; 3 Departments of Neurosurgery and Physiology, Loma Linda University, Loma Linda, CA, USA

**Keywords:** Xenon, Hypoxia, Ischemia, Neuroprotection, Anesthesia, Hypothermia, NMDA

## Abstract

Xenon is a medical gas capable of establishing neuroprotection, inducing anesthesia as well as serving in modern laser technology and nuclear medicine as a contrast agent. In spite of its high cost, its lack of side effects, safe cardiovascular and organoprotective profile and effective neuroprotective role after hypoxic-ischemic injury (HI) favor its applications in clinics. Xenon performs its anesthetic and neuroprotective functions through binding to glycine site of glutamatergic N-methyl-D-aspartate (NMDA) receptor competitively and blocking it. This blockage inhibits the overstimulation of NMDA receptors, thus preventing their following downstream calcium accumulating cascades. Xenon is also used in combination therapies together with hypothermia or sevoflurane. The neuroprotective effects of xenon and hypothermia cooperate synergistically whether they are applied synchronously or asynchronously. Distinguishing properties of Xenon promise for innovations in medical gas field once further studies are fulfilled and Xenon’s high cost is overcome.

## Introduction

**Figure 1:**
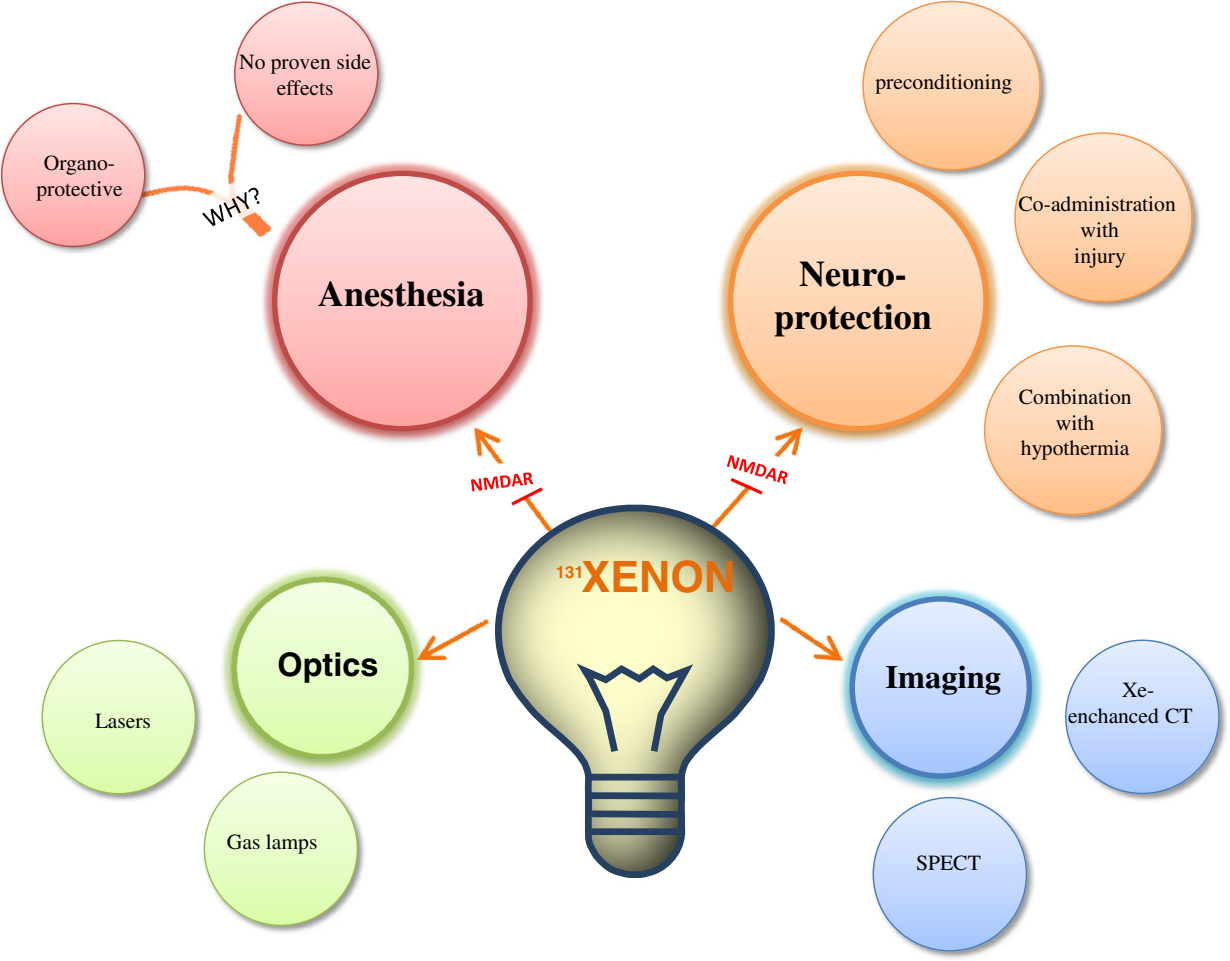
**Schema Summarizing Xenon**’**s applications**: **Noble gas xenon is used in various fields.** In medicine, Xenon can be utilized as a neuroprotective and anesthetic agent. It can also be used as a contrast agent in imaging. Plus it has the potential to be used in the field of optics.

Medical gases have a wide scope of applications in medical area. In the field of medicine, medical gases are used for many practices such as anesthesiology, hyperbaric oxygen therapy, neuroprotection and hypothermia [1]. For instance, hydrogen, the first element in periodic table, is a medical gas that is synthesized in fermentation of non-digestible carbohydrates in human cells [1-3], has a neuroprotective role following middle cerebral artery occlusion (MCAO) [4], neonatal HI [5,6] and newborn pig asphyxia models [1,7]. Helium which has the second highest prevalence in the universe [1] has therapeutic effects on arrthymia [8] and inflammation [9,10], and has myocardioprotective role [1,11]. Argon, another noble gas has been used as anesthetic and neuroprotective agent [12]. Xenon which is a medical trace gas in Earth’s atmosphere [13] is proven to be effective in the fields of anesthesia and neuroprotection [12] (Figure 1).

In vitro and in vivo studies prove that Xenon has therapeutic effects on various neurodegenerative outcomes. Due to these promising results, in this paper we aimed to review the beneficial applications of Xenon in clinical aspects mainly for neuroprotection as well as anesthesia.

### Discovery and chemical properties of xenon

In 1898, Sir William Ramsay who was a Professor of Chemistry at University College London discovered Xenon. Ramsay, a Noble prizewinner for the discovery of krypton and neon, also found Xenon with his student Morris Travers in the residue of evaporating components of liquid air [14,15]. Today, Xenon is still purified by fractional distillation of liquefied air; similar to the method Ramsay had performed [15]. With his findings, Ramsay estimated that Xenon was found in 1 part in 20 million in Earth’s atmosphere [14]. Recent studies prove the occurrence of Xenon that Ramsay had estimated [15].

After Xenon’s discovery in 1898, Albert R. Behnke Jr. used different breathing mixtures including Xenon in his studies of exploring the drunkenness in deep sea divers [16]. He concluded that Xenon could be used as an anesthetizing agent with the results obtained from his trials in 1939. Following this outcome, Xenon was used as a surgical anesthetic agent for the first time by Stuart C. Cullen and Gross in 1951 [16,17]. It was selected to be an effective anesthetic with its low blood-gas partition coefficient (0.115) [14,18,19], safe cardiovascular profile [17] and ability to penetrate through blood brain barrier without extensive effort [14,17,18]. These advantageous properties enable Xenon to have a rapid induction, which is a key element in anesthesia [1,17]. Therefore, among the first five noble gases in the periodic table that are helium, neon, argon, krypton and Xenon, Xenon is the most potent in anesthesia [20]. Moreover, Xenon is non-teratogenic [14] and non-fetotoxic [15,21]. Hence, it is a good interest for research with its possible neuroprotective and anesthetic properties.

Xenon is a colorless, heavy and odorless noble gas [1]. Having the chemical properties of noble gases, Xenon cannot form covalent bonds with other molecules. However, it can still have binding action via van der Waals forces. These forces occur in instantaneously polarized atoms. This can be the only mechanism of binding, since noble gases are non-polar and uncharged. Spontaneous polarization enables the formation of a charged binding site of the atom so that it can attract surrounding molecules. In Xenon’s case, spontaneous polarization of Xenon helps it bind the active sites of enzymes and receptors by interfering with amino acids [12,22]. Thanks to its high number of electrons and lower-binding energy, Xenon’s ability to be polarized is greatest among other noble gases [12].

### Neuroprotective role of xenon

Cardiac arrest (CA) is one of the major causes of death in both United States and Europe [23,24]. Although with advanced and adventitious cardiopulmonary resuscitation systems, almost 50% of CA victims are saved [25,26], the next threat for resuscitated patients is global cerebral hypoxia-ischemia [27,28]. It increases the mortality after sudden CA with the mechanism of necrosis or apoptosis of neuronal tissue [29-31]. Cognitive dysfunction, a permanent or transient inability to perform daily activities [32,33] is an expected result, especially due to hippocampal injury [34-36].

Another major risk group that is affected by hypoxia-ischemia is neonates, because of the devastating nature of labor procedure. Up to 1-2/1000 live births result in severe hypoxia-ischemia and 60-70% of the ones suffering from hypoxia-ischemia may die or suffers life-long disabilities [37]. The risk of having hypoxia-ischemia after CA or in perinatal period cannot be anticipated but should be scrutinized. Since the risk of having hypoxia-ischemia is unpredictable, it is unlikely to develop a method to be administered prior to insult [19]. Consequently, a therapeutic strategy or a neuroprotective agent should be evolved to manage and limit the effects of hypoxia-ischemia after it eventuates [17].

#### In-vivo studies

**Figure 2:**
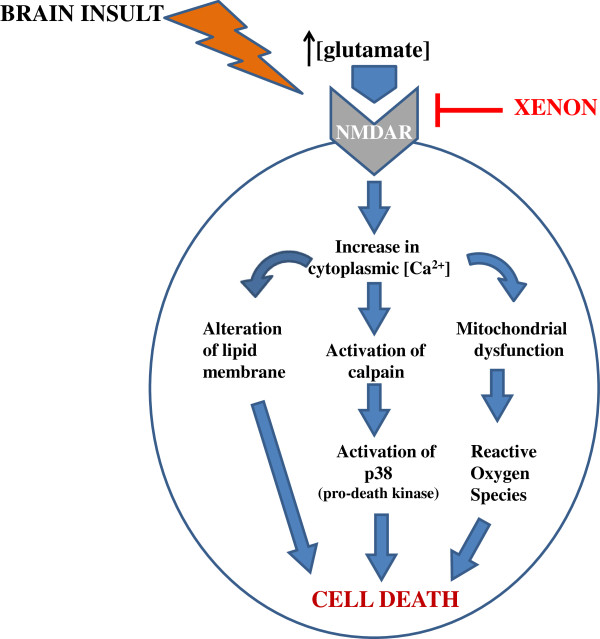
**Mechanism of Xenon**’**s neuroprotective action**: **The underlying mechanism of action which Xenon exerts its neuroprotective effect is based on blockage of NMDA receptors.** Without Xenon blockage, increase in cytoplasmic calcium concentration due to overstimulation of NMDA receptors leads to cell death by various pathways. Xenon inhibits these cascades by competitively binding to glycine site of NMDA receptors.

Recent in vivo studies on sub-anesthetic dosage of Xenon with 50% concentration had promising results against neonatal hypoxia-ischemia [1], CA induced cerebral ischemia [36] and neurobehavioral dysfunction caused by brain insult [38]. Major mechanisms lying behind Xenon’s neuroprotective property are the reduction of ischemia-induced neurotransmitter release [39] as well as the antagonistic property against NMDA receptors [36,37,40] (Figure 2). It has been proven by Franks and his colleagues that Xenon’s property of being antagonist to NMDA receptors is a key factor [41], since NMDA receptors are primarily involved in initiation and progression of apoptosis in neural tissue [19,42]. Fries et al. state that after Xenon treatment, there was a reduction of perivascular inflammation in putamen and caudate nucleus on pigs with post CA [36]. In addition, Dingley et al. showed short-term neuroprotective effects of Xenon administration in neonatal rats which were exposed to hypoxia-ischemia [1,43]. Also, it was demonstrated that preconditioning of Xenon displays neuroprotective effects like reducing the size of infarction and enhancing neurological functions in neonatal rats having hypoxia-ischemia [1,44]. It has also been indicated that Xenon has a higher efficacy in cortex rather than subcortex due to the difference in vascularity and in density of NMDA receptors in two distinct layers of cerebrum [45].

Another beneficial property of Xenon is its non-toxic chemical characteristic which enables the usage of the gas on neonates. The high risk of any traumatic event during childbirth brings the idea of Xenon preconditioning to mother as a method of reducing the possibility of brain insult to the neonate [46]. Xenon is an ideal gas for this model with its cardiovascular stability [47] and myocardial protection property [48], as well as its rapid induction rate through blood–brain-barrier [14,49]. As a result of these favoring characteristics, clinical usage of Xenon for neuroprotection was approved in Europe in 2007 [50,51].

Focal cerebral ischemia, in which a blood clot occludes blood vessels of the brain [52], is commonly induced by in-vivo animal model called filament occlusion of middle cerebral artery [45]. It has been indicated that application of Xenon during MCAO in mice reduces total infarct size and augments neurologic outcome [18,19,45]. Besides its co-administration with the injury, its preconditioning also provides neuroprotection in mice model of MCAO [53].

#### In-vitro studies

Oxygen glucose deprivation (OGD), which is one of the in vitro models of ischemia, is a condition that neurons undergo when cerebral blood flow is disrupted. This deprivation activates excitoxicity in which NMDA glutamate receptors are over stimulated, leading to neuronal death through apoptosis and necrosis [18]. It has been shown that Xenon reduces neuronal injury by its administration before and during the insult [20,44,54]. Bantel et al. demonstrated neuronal preconditioning property of Xenon by exposing neuronal-glial co-cultures to 75% Xenon for 2 hours [55] as illustrated in Table 1. Another study that was held by Maleeha et al. showed that Xenon preconditioning protects human kidney cells from OGD injury. They proposed the mechanism in which survival factors p-Akt, HIF-1a and Bcl-2 are upregulated [20].

**Table 1:** **In**-**vivo and in**-**vitro models demonstrating the neuroprotective effects of Xenon**

**Reference**	**Model**	**Intervention**	**Results**
Bantel et al., 2009	Neuronal damage due to OGD	Preconditioning with 75% Xenon for 2 hours	Xenon preconditioning reduces neuronal injury through activation of plasmalemmal ATP sensitive potassium channel [62]
Liu et al, 2011 Wilhelm et al, 2002	Neuronal damage due to OGD	Xenon saturated medium for 24 hours (in vitro)	Reduction in lactate dehydrogenase release proves that Xenon reduces OGD induced neuronal cell death at subanesthetic concentrations [1,61].
Homi et al, 2003	MCAO in mice	70%, 35% Xenon during middle cerebral artery occlusion for 1 hour	Application of Xenon during MCAO reduces total infarct size and augments neurologic outcome [54].
Horiguchi et al, 2006	MCAO in mice	Preconditioning with 70% xenon for 2 hours	Xenon preconditioning induces neuroprotection in mice model of middle cerebral artery occlusion [64].
Hobbs et al, 2008	Neonatal HI	Hypothermia (32°C) with inhalation of 50% Xenon for 3 hours	Combination of hypothermia and Xenon increases neuroprotection from 37% (hypothermia only) to 76% [70].
Martin et al, 2007	Neonatal HI	Asynchronous application of Xenon (20%) and hypothermia (35°C) with 1 hour and 5 hours gap in between the treatments	Asynchronous combination of Xenon and hypothermia reduces brain infarction significantly [71].
Ma et al, 2006 Luo et al, 2008	Neuronatal damage due to OGD	Combination of 20% Xenon and 0.75% sevoflurane preconditioning	Combination of Xenon and sevoflurane preconditioning induces long term neuroprotection by enhanced phosphorylated cyclic adenosine monophosphate response element binding protection signaling. [53,55]

In oxygen glucose deprivation induced neuronal damage and middle cerebral artery occlusion in mice models, both Xenon preconditioning and its co-administration with insult grant neuroprotection. Combinations of Xenon with hypothermia in neonatal hypoxia-ischemia model and with sevoflurane in oxygen glucose deprivation induced neuronal damage model also enhance neuroprotection.

Xenon also acts as a neuroprotectant when co-administered with OGD injurious agents. It has been shown that Xenon reduces OGD induced neuronal cell death in mouse neuronal-glial cell co-culture at subanesthetic concentrations. Since lactate dehydrogenase release is an indication for cell injury, Xenon’s mentioned neuroprotective role has been assessed by the reduction in lactate dehydrogenase release [1,54]. Helene et al. also proved that Xenon reduces lactate dehydrogenase and dopamine release, which is a key event in excitotoxicity in OGD [18]. This again shows that Xenon attenuates neuronal injury when it is co-administered with OGD.

#### Combination therapy studies

Currently, hypothermia is the only intervention that provides neuroprotection after hypoxia-ischemia [37,56-58]. It has been proven that it enhances neurological outcome after hypoxia-ischemia not only in neonatal rats, pigs, and sheep but also in infants who suffer from hypoxic ischemic encephalopathy [14] and adults after out-of-hospital CA [59]. Hypothermia makes it possible for one in six infants with neonatal encephalopathy to have reduced neurological deficits [58,59]. In order to increase this ratio, drugs which are able to enhance hypothermia’s neuroprotection should be investigated. Xenon is a promising candidate for this role because of its lack of chemical reactivity, lack of side effects, non-fetotoxicity, and easy reversibility [37].

Studies have shown that Xenon augments neuroprotection when combined with hypothermia in animal hypoxia-ischemia models [43,60]. Hobbs et al. have demonstrated that a combination therapy of hypothermia (32°C) with inhalation of 50% Xenon for 3 hours increases neuroprotection from 37% (hypothermia only) to 76% (hypothermia combined with xenon inhalation) [60,61]. The synergistic neuroprotective effect of this treatment is still obtained even after asynchronous administration of Xenon and hypothermia. When Xenon (20%) and hypothermia (35°C) were applied asynchronously with 1 hour and 5 hours gap in between the treatments, their neuroprotective effect combined synergistically and brain infarction in neonatal rat hypoxia-ischemia model was reduced significantly [62].

Hypothermia decreases the release of glutamate that binds to NMDA receptors. It also reduces the release of glycine which assists glutamate to act on the NMDA receptor. Since Xenon is a NMDA receptor antagonist, hypothermia’s role of decreasing neurotransmitter and Xenon’s role of receptor blockage converge on an anti-apoptotic pathway [61]. This mechanism might also explain why asynchronous administration of Xenon and hypothermia still improves the neurologic outcome of hypoxia-ischemia [14].

It has also been studied whether a combination therapy of Xenon with other drugs like dexmedetomidine and sevoflurane is effective against hypoxia-ischemia. Combination of Xenon with dexmedetomidine reduces OGD induced brain infarction in vivo [63]. Also, OGD injury is decreased in vivo by independent preconditioning of Xenon and sevoflurane [44,46]. Yan et al. have shown that combination of Xenon and sevoflurane preconditioning induces long term neuroprotection in neonatal asphyxia. This combination gives the chance to apply Xenon in lower doses, thus to decrease its high cost [46].

### Mechanism of action

Xenon is an NMDA receptor antagonist [41]. It competitively binds to the same site as NMDA receptor co-agonist glycine [64] by interacting with the aromatic ring of phenylalanine 758 [65]. Inhibition of NMDA receptors by this binding enables Xenon to perform its anesthetic and neuroprotective roles [41,64].

NMDA receptor is an ionotropic receptor that is activated by binding of glutamate [66]. Although glutamate is the main excitatory neurotransmitter of central nervous system, its excessive or constant activation of NMDA receptor causes neuronal death after ischemia or stroke due to increase in cytoplasmic calcium concentration [66-70]. Influx of calcium through the stimulated NMDA receptor increases the nitric oxide (NO) production and cell membrane lipid peroxidation. NO, a free radical, is a potent vasodilator and is synthesized by NO synthase in endothelial cells and neurons in response to rise in intracellular calcium. Excessive generation of NO causes mitochondrial dysfunction. NO and superoxide (O_2_^–^) react, resulting in large amounts of peroxynitrite (ONOO^-^) generation. Peroxynitrite causes DNA damage via oxidative reactions [71]. Additionally, increased calcium concentration results in activation of calpain, which in turn activates p38 (pro-death kinase), activation of stress-induced protein kinase (such as p38 and JNKs) and aggregation of proteins and nucleic acids that deteriorates lipid bilayer membrane [72] resulting in cell death. Xenon blocks NMDA receptor at the glycine binding site [73] and inhibits these excitotoxic pathways, thus establishes neuroprotection [74].

In addition, Xenon, like general anesthetics, is known to produce hypothermic effect which is also neuroprotective [74]. Hypothermia reduces excitotoxicity by decreasing glutamate and glycine release [61] and interfering with formation of reactive oxygen species [75].

### Anesthesia

The use of modern anesthesia was introduced by Dr. William Morton in 1846 [76] during a dental surgery with inhalation of ether and the word ‘anesthesia’ was introduced by Dr. Oliver Wendell Holmes in the same year [77]. In general anesthesia, most preferred drugs are volatile anesthetics which are structurally related to ethers. These molecules are relatively more soluble in lipids than in water, so it is believed that their primary site of action is membrane proteins of nerve cells [78]. Recently there has been a renewed interest in one of the noble gases, Xenon, because of its cardio protective profile. On controversy to halogenated ethers, Xenon anesthesia has no harm on cardiovascular system furthermore it has a neuroprotective effect [79]. Yet there is still limitation in its widespread application due to its highly costs.

Although Xenon was declared to be a possible anesthetizing agent in 1939 by Albert R. Behnke Jr, it took 7 more years to be proven that Xenon has anesthetic properties by the publication of John H. Lawrence [80]. Five years had passed after the first publication to be used in a human surgery that was accomplished by American anesthesiologists Stuart C. Cullen in 1951 [45,80]. Till 1990s, Xenon was not a gas which was intensively researched on [81]. However, in last decades the popularity of Xenon increased due to its harmless and beneficial profile. Russia was the first country to approve the application of Xenon in anesthesia in 2000. Germany was the next country to authorize in 2005. In 2007, the approval for the usage of Xenon as an anesthetic agent was extended to all Europe [80,82].

Xenon is claimed to be an ideal anesthetic due to its unique properties [19,81,82]. It was observed that patients receiving Xenon anesthesia have a shorter recovery time and reduced post-operative pain [81] when compared to patients exposed to other volatile anesthetics [19]. The reason underneath is Xenon’s low blood-gas partition coefficient [83].

It was also proven that Xenon doesn’t have any side effects to human body [81,84,85]. It has been shown in vitro that Xenon doesn’t cause any coagulation [86] or platelet dysfunction [87] and it doesn’t deteriorate hepatic or renal functions [88,89]. Besides its organoprotective property, it demonstrates a low blood-gas partition coefficient of 0.115 and fast diffusion rate through blood brain barrier which enables Xenon to be both inducted and washed out in a short amount of time [13]. Xenon has further advantages like preferred hemodynamic stability [80] and rapid perfusion to specific organs [16]. As a result, Xenon is correlated with maintaining a slower heart rate and a greater arterial pressure value than other anesthetics [90]. This adventitious cardiovascular profile enables the usage of Xenon as an anesthetic agent during the operations on patients having coronary artery disease with tachycardia and low arterial pressure that are life threatening for them [91].

Other than being non-hazardous to cardiovascular system, Xenon can be also used as an anesthetic agent because of its additional neuroprotective role [41,92]. Most general anesthetics induce anesthesia by potentiation of gamma-amino butyric acid receptor which is an inhibitory synaptic receptor [77]. Xenon, on the other hand performs its anesthetic role by blocking NMDA receptor similar to its neuroprotective mechanism [1,41]. This blockage inhibits excitatory neurotransmission of NMDA receptors, thus causes anesthesia. Franks et al. proved that 80% Xenon decreased NMDA-activated currents by approximately 60%. Xenon’s blockage of NMDA receptors enables it to have anesthetic effects, since NMDA receptors are involved in synaptic mechanisms of perception of pain, learning and memory [41]. It is claimed that Xenon induces anesthesia also by activating two pore domain background potassium channel TREK-1 [19]. Once activated, these channels cause neuronal hyperpolarization. Consequently, cellular excitability is decreased and anesthesia is provided [93,94].

Besides NMDA receptor, the other two glutamate receptors are α-amino-3-hydroxy-5-methyl-4-isoxazolole propionate (AMPA) receptor and kainate receptor which are also called as “non-NMDA receptors”. Since the structures of these three glutamate receptors are very similar to each other, whether AMPA and kainate receptors are additional molecular targets for Xenon has been investigated [39]. Most studies confirm that Xenon causes anesthesia by inhibiting NMDA receptors, whereas the studies on its effects on AMPA and kainate receptors are contradictory [95]. In a study performed by Plested et al., AMPA receptor was expressed in *Xenopus* oocytes and HEK-293 cells. Its sensitivity to Xenon and how it was affected by desensitization was observed by bath application of Xenon and the recording of the resulting ionic currents. When kainate was used as an artificial agonist, Xenon blocked AMPA receptor due to desensitizing response elicited by kainate. In the second part of the study, glutamate which is a natural agonist of AMPA receptor was used rather than kainate by an ultra-rapid application system which mimicked the rapid release of high-concentration glutamate at synapses. This time Xenon didn’t block AMPA receptor due to lack of desensitization. Therefore, it was concluded that the sensitivity of AMPA receptor to Xenon depended on desensitization and that AMPA receptor was not blocked during anesthesia with Xenon [96].

Dinse et al., on the other hand, demonstrated by using voltage-clamped cortical neurons from embryonic mice that desensitization still occurred with the fast application of glutamate. Xenon blocked AMPA and kainate receptors even when glutamate was used as the agonist, so it was concluded that the blockage of AMPA and kainate receptors in cortical neurons contributed to Xenon’s anesthetic property [39]. Georgiev et al. agreed that Xenon performed its anesthetic action by blocking both NMDA and AMPA receptors but they claimed that this blockage occurred in spinal cord dorsal horn neurons rather than cortical neurons [95]. When these contradictory studies are examined, it is seen that the role of AMPA and kainate receptors in Xenon’s anesthetic action is not as clear as the role of NMDA receptor [39]. Therefore, further investigations are needed for enriching Xenon’s anesthetic mechanisms.

### Use of xenon in optics

In the field of optics, noble gas Xenon is used for various purposes. After its discovery, Xenon was used in flash lamps for the first time for photography [97]. Today the majority of cameras still use Xenon gas in modern flash technology. Many of the gas-discharge lamps that we use in our daily lives like car bulbs and ceiling lights contain Xenon for energy-efficient illumination as well.

Xenon compounds are also used commonly in modern laser technology. Excimer lasers that are used for photolithography and laser eye surgery contain Xenon gas [98]. Under appropriate conditions of high pressure and electrical stimuli, Xenon gives rise to laser light in ultraviolet range by forming a compound with other excitable molecules. This relatively cheap but easy method makes it possible to selectively remove micrometer size films for producing computer chips and provide treatment to people who suffer from myopia, hyperopia and astigmatism [99].

Since Xenon flash lamps have an emission spectrum ranging from ultraviolet to infrared light, these lamps are also used in food industry and dermatological practices. In food industry, Xenon flash lamps are designed in a way that they provide high power pulses of light for killing microorganisms including bacteria, yeasts, molds and viruses [100-102]. This treatment is approved by Food and Drug Administration (FDA) in United States since it is an effective thermal treatment for food decontamination. In dermatologic practices, intense pulsed light is used for removal of hair and photo-rejuvenation [103]. Once applied to skin, light beams at a specific wavelength will target bulbs of hair where melanin pigments are abundant. The heat generated by the absorbed light will vaporize hair bulbs, which will lead to hair reduction in skin. The same principle also applies for removal of skin lesions. By using specific wavelengths and intensity of light, photo-rejuvenation therapies can remove pigmented lesions [104]. This is achieved by harnessing the efficacy of monochromatic excimer light [105].

### Medical imaging

With the last technological advancements in nuclear medicine; different methods were developed in recent years aiming for better results. The idea of using Xenon gas was introduced lately in computed tomography (CT) - imaging as well as single-photon emission computed tomography (SPECT) since it has some advantages over other techniques that are currently being used [106].

Xenon enhanced CT scanning is an easy and non-invasive method in which patients inhale the stable (^131^Xe) gas while CT images are made. It can be used for various findings like measuring cerebral blood flow after a traumatic brain injury. When clinical urgency of a traumatic patient is considered, Xenon enhanced CT is a fast and reliable procedure since less than 15 minutes of gas inhalation is required for the process. In this method, non-radioactive molecule Xenon (^131^Xe) is used as a contrast agent [107], for getting good resolution images [108]. It is non- hazardous for the body since half-life of ^131^Xenon is about 40 seconds in cerebral tissue so it is washed away rapidly after inhalation [109]. Once inhaled, diffusion rate of the gas into tissue would indicate the amount of blood flow that area is receiving, so that further clinical examinations can be administered. This technique is also used for measuring tissue blood flow in pancreatic tumors [110].

In SPECT, radioactive isotope (^133^Xe) of Xenon is used as radionuclide. Since Xenon is a small inert molecule, soluble in lipid and water, readily exchanged between blood and tissue, and diffusible in the brain, it is considered as a good agent for SPECT [105]. This technique can be used for obtaining quantitative and repeatable whole-brain scans [111], ventilation studies of lungs, measurement of cerebral blood flow [107] to study pathology of dementia, Alzheimer's disease, epilepsy, obsessive-compulsive disorders [112].

### Toxicity

Volatile and inhalation anesthetics are dangerous for ozone layer because of their chlorofluorocarbon based structures [19]. One of the inhalational agents, nitrous oxide, is 230 times more potent as a greenhouse gas when compared with carbon dioxide [113]. On contrary, Xenon has no undesirable ecological effects since it is a naturally occurring gas in Earth’s atmosphere.

Xenon is a good candidate for being an ideal anesthetic not only because of its beneficial effects, but also its lack of toxicity. Besides being non-neurotoxic, Xenon doesn’t have any effect on hematological and biochemical variables as well [88]. It has been shown that Xenon has no impact on coagulation/platelet function [87], nor on immune system [114]. Xenon is non-reactive in the body, it is disposed from the lungs without interfering with hepatic and renal systems, thus it doesn’t impair hepatic or renal function [88,89].

### Cost-efficiency

Xenon is a trace element in Earth’s atmosphere [60], expense of its fractional distillation is very high and it cannot be industrially synthesized [50]. Therefore, Xenon is relatively more expensive than other anesthetic and neuroprotective agents [115]. Xenon’s scarcity and high cost (10$/L) [85] are major handicaps along the way of being used commonly as an ideal anesthetic and neuroprotectant [60]. While other volatile anesthetics have a cost around 10$ for 2 hours of administration, Xenon’s expense for the same time interval is 300$ in total [90]. Consequently, cheaper methods should be investigated to use Xenon more efficiently and economically. A closed-circulatory system is recently developed for this scope [18], enabling the reuse of Xenon gas and mechanical replenishment of oxygen [61]. On the other hand, the high cost of Xenon might not be as great as it seems, since Xenon anesthesia improves clinical outcome. Reduced hospital stay duration and decreased need of post-operative intensive care might outweigh the cost of Xenon [90]. It seems further studies are necessary to evaluate cost effectiveness of Xenon in clinical usage.

## Conclusion and further studies

Xenon has a bright future in medicine due to its noticeable advantages over other anesthetic agents. Despite of its highly costs, its neuro and myocardio protective profile, non-toxic chemical properties, nature friendly feature and efficacy in hypoxia-ischemia treatment combined with hypothermia makes it an ideal candidate for innovations in medical gas field. Promising findings obtained from in vivo-vitro studies of xenon denote that xenon will take its part in neuroprotective treatments for brain trauma, resuscitation and ischemic insults. However, further research and large-scale investigations are necessary for utilizing Xenon’s therapeutic potential maximally. The highly cost of Xenon should also be overcome for achieving this goal.

## Abbreviations

HI: Hypoxic-ischemic injury; MCAO: Middle Cerebral Artery Occlusion; FDA: Food and Drug Administration; CA: Cardiac Arrest; NMDA: N-methyl-D-aspartate; OGD: Oxygen glucose deprivation; NO: Nitric Oxide; O2: Superoxide; ONOO: Peroxynitrite; AMPA: α -amino-3-hydroxy-5-methyl-4-isoxazolole propionate; CT: Computed Tomography; SPECT: Single-photon emission computed tomography.

## Competing interests

The authors declare that they have no competing interests.

## Authors’ contributions

EE, SY, YB, and AR identified the subject, did literature search and wrote the initial draft, IS revised the manuscript, JHZ helped to identify the subject and revised the manuscript. All authors read and approved the final manuscript.
